# Surface Modification of Nanopores in an Anodic Aluminum Oxide Membrane through Dopamine-Assisted Codeposition with a Zwitterionic Polymer

**DOI:** 10.1021/acs.langmuir.3c03654

**Published:** 2024-02-26

**Authors:** Chien-Wei Chu, Chia-Hsuan Tsai

**Affiliations:** Department of Chemical Engineering, Feng Chia University, Xitun District, Taichung 40724, Taiwan

## Abstract

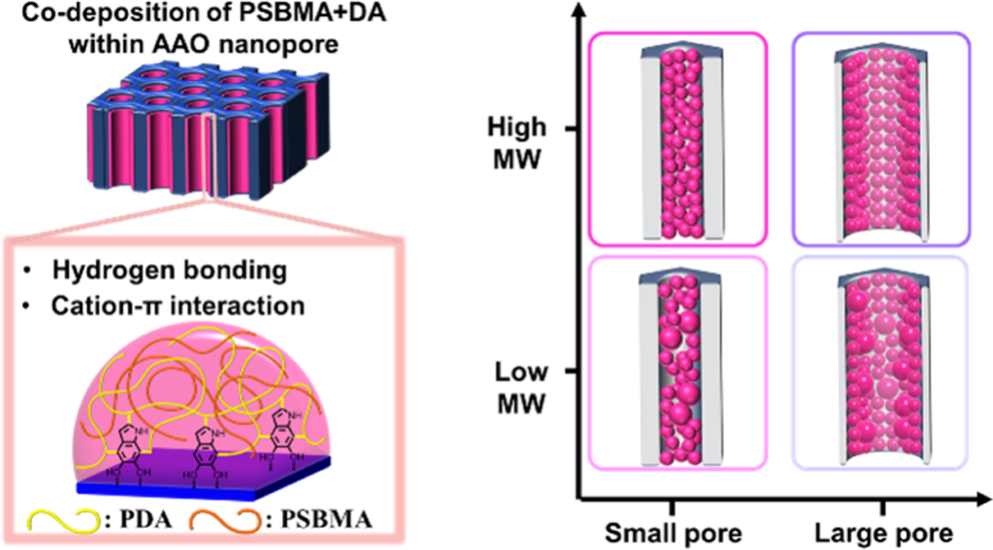

Surface modification through dopamine-assisted codeposition with functional zwitterionic polymers can provide a simple and one-step functionalization under ambient conditions with robust and stable dopamine–surface interactions to improve the hydrophilicity of nanoporous membranes, thereby expanding their applicability to nanofiltration, ion transport, and blood purification. However, a significant knowledge gap remains in our comprehension of the mechanisms underlying the formation and deposition of dopamine/polymer aggregated coatings within nanoscale confinement. This study explores a feasible method for membrane modification through the codeposition of dopamine hydrochloride (DA) and poly(sulfobetaine methacrylate) (PSBMA) on nanopores of anodic aluminum oxide (AAO) membranes. Our findings demonstrate that the aggregated coatings of DA and PSBMA nanocomposites can effectively deposit on the surfaces within cylindrical AAO nanopores, significantly enhancing the hydrophilicity of the nanoporous membranes. The morphology and homogeneity of the nanocomposite coatings within the nanopores are further investigated by varying PSBMA molecular weights and AAO pore sizes, revealing that higher molecular weights result in more uniform deposition. This work sheds light on understanding the codeposition of DA and zwitterionic polymers in nanoscale environments, highlighting a straightforward and stable surface modification process of nanoporous membranes involving functional polymers.

## Introduction

In recent decades, nanoporous membranes have emerged as versatile platforms with applications across various fields, including biomedical sensing,^1,2^ nanofiltration,^3−5^ energy conversion,^6−8^ and smart separation and gating.^9−11^ The nanoporous membranes, exhibiting high performance and versatility, usually feature well-controlled nanopore geometries and advanced materials with excellent functionality.^8,12,13^ Achieving both of these two key characteristics simultaneously often involves surface modification of nanoporous membranes using functional polymer materials, which proves to be a powerful and effective strategy.^14−16^ Numerous surface modification methods with polymers have been developed based on various interfacial interactions. For example, a simple deposition method is frequently utilized to modify nanoporous membranes by dip or spin coating using polymer solutions, capitalizing on physical interfacial bonding, such as van der Waals forces or hydrogen bonding.^17,18^ The layer-by-layer assembly method has also been employed for membrane modification through electrostatic interactions with charged polymers.^19,20^ Additionally, the surface-initiated polymerization method has been introduced to modify membranes with high-grafting density and homogeneous polymer brushes through covalent bonding.^21−23^ These mentioned traditional surface modification methods, however, usually suffer from issues such as weak bonding, the need for specialized polymer and membrane materials, or complex polymer synthesis techniques, which can limit their applicability for long-lasting usage and mass production development.

Inspired by the robust adhesion behavior of mussels to universal substrates,^24^ biomimetic chemicals containing dopamine/catechol structures have been widely introduced in recent years for surface modification.^25−28^ One such approach is the dopamine-assisted codeposition with polyelectrolytes (e.g., polyzwitterions), which has proven to be highly effective in forming specific interactions with desired polyelectrolytes. These dopamine–polyelectrolyte interactions may include hydrogen bonding, cation–π interactions, and covalent bonding through the catechol-amino Schiff base reaction and Michael addition.^29^ The codeposition mechanism involves the formation of composite assemblies of cross-linked polydopamine (PDA) and polyelectrolytes, followed by the robust anchoring of desired polyelectrolytes to various types of substrates.^29−31^ By incorporating dopamine-containing chemicals, surface modification with polymers can be achieved through one-step functionalization under ambient conditions, mimicking the mussel’s adhesive properties with stable dopamine–surface interactions. This sheds light on the development of simple modification techniques to expand the applicability of functional nanoporous membranes.

Dopamine-assisted codeposition with various polymers, such as poly(sulfobetaine methacrylate) (PSBMA), has been extensively employed in nanoporous membrane modification to improve hydrophilicity, ion selectivity, biocompatibility, and antifouling property.^32−36^ The enhanced properties, particularly hydrophilicity, can significantly boost their performance in applications such as water purification, ion separation, blood purification, and optical anisotropy.^32−37^ A notable gap, however, still exists in our understanding of the processes involved in forming and depositing dopamine/polymer aggregated coatings within nanoscale spaces. In this work, we present a method for modifying nanoporous anodic aluminum oxide (AAO) membranes by codepositing PSBMA and dopamine hydrochloride (DA) within the AAO nanopores. We successfully demonstrated surface modification on alumina-based surfaces, both on flat alumina substrates and nanoporous AAO membranes. Increased hydrophilicity, as determined through contact angle measurements, along with elemental analysis using X-ray photoelectron spectroscopy (XPS), chemical structure identification via Fourier-transform infrared spectroscopy (FTIR), and ultraviolet–visible (UV–vis) spectroscopy, confirmed the presence of PSBMA and its interaction with DA. Codeposition within the AAO nanopores was further confirmed through scanning electron microscopy (SEM) and XPS with specialized cross-sectional holders. Effects of molecular weights of PSBMA and pore sizes of AAO membranes on aggregated PDA/PSBMA coating morphology and homogeneity within nanoscale spaces were also investigated. The results indicate that higher molecular weights lead to a more uniform modification. This study offers a deeper insight into the codeposition of DA and the polyzwitterion within the nanoscale spaces and highlights a straightforward membrane modification process with functional polymers.

## Results and Discussion

### Surface Modification with the Polyzwitterion through Dopamine-Assisted Codeposition

**Figure 1:**
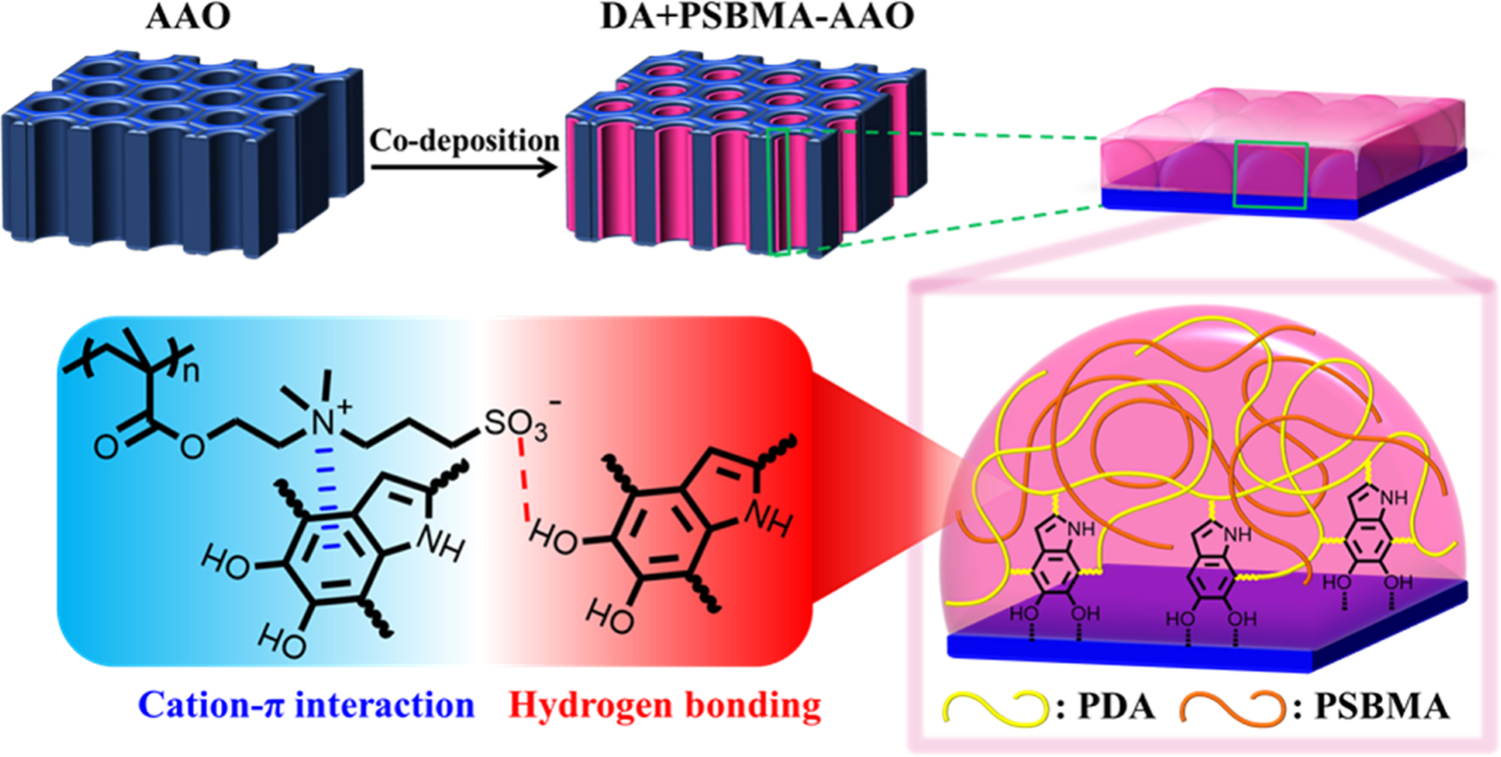
Scheme illustration of surface modification of AAO membranes with PSBMA via dopamine-assisted codeposition. The DA + PSBMA composite coating is composed of aggregated PDA/PSBMA nanoparticles. The PDA moiety provides a strong catechol–oxide interfacial interaction with the AAO surface for robust immobilization. It can also adsorb the polyzwitterion via the cation−π interaction, hydrogen bonding, and chain entanglement to exhibit the functional surface properties of PSBMA.

A straightforward and effective method for modifying nanoporous membranes to give them improved hydrophilic properties was proposed and demonstrated through the codeposition of poly(sulfobetaine methacrylate) (PSBMA) and dopamine hydrochloride (DA) within the nanopores of anodic aluminum oxide (AAO) membranes (Figure 1). The detailed process of surface modification is described in the Experimental Section. The resulting coating layer on the cylindrical nanopore surfaces of the modified AAO membrane, called the DA + PSBMA-AAO membrane, consists of aggregated nanoparticles of PSBMA and self-polymerized polydopamine (PDA).^29−31^ The PDA component not only provides robust bonding to anchor the coating onto the AAO surfaces, mimicking the mussel’s bioadhesion functionality,^24^ but also forms specific interactions with PSBMA polymer chains, including ammonium cation–π interactions, hydrogen bonding, and chain entanglement.^29−31^ This ultimately can endow the AAO membranes with the desired functional surface properties, such as hydrophilicity, from the PSBMA polymers. The various PSBMA polymers with different molecular weights (*M_n_*: 18.4, 56.2, and 106.3 kg/mol) and narrower molecular weight distribution (*Đ* < 1.21), intended for use in further mechanistic studies on the codeposition process, were synthesized through reversible addition–fragmentation chain-transfer (RAFT) polymerization and well identified using nuclear magnetic resonance (^1^H NMR) spectroscopy, thermogravimetric analysis (TGA), and gel permeation chromatography (GPC) (Figure S1). The measured characteristics are consistent with those reported in the previous literature for PSBMA.^21,38^

### Verification and Duration Optimization for the Codeposition Process on Flat Substrates

**Figure 2:**
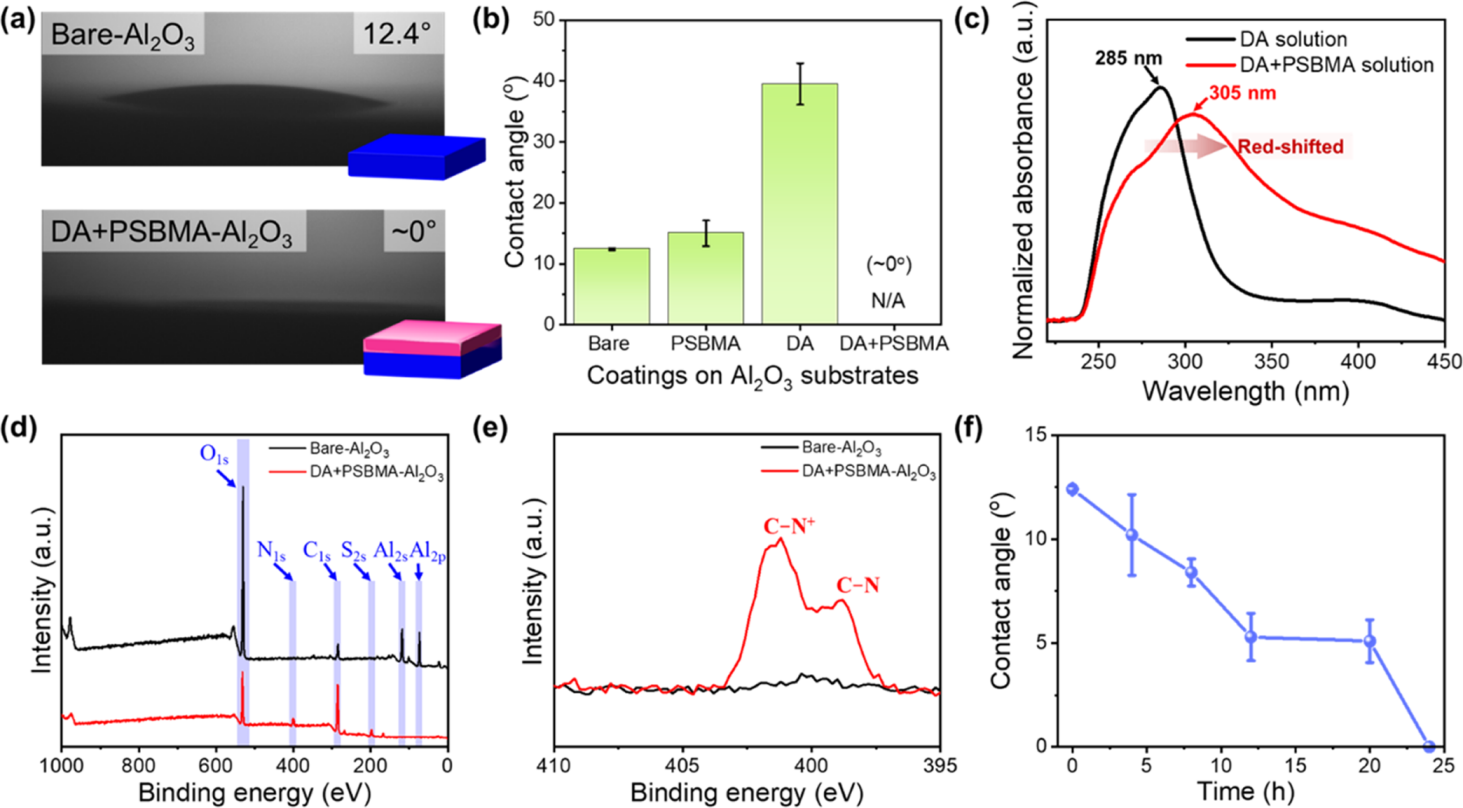
Surface characterizations of bare and modified flat Al_2_O_3_ substrates. (a) Water contact angles on bare-Al_2_O_3_ (12.4°) and DA + PSBMA-Al_2_O_3_ (∼0°). (b) Comparison of water contact angles of bare, PSBMA-coated, DA-coated, and DA + PSBMA-coated Al_2_O_3_ substrates after ultrasonication washing. (c) UV–vis spectra of the diluted DA and DA + PSBMA solutions incubating in the air for 4 h. An additional red-shifted peak at 305 nm in the DA + PSBMA solution implies the delocalization of the π-electron in dopamine induced by the formation of the cation–π interaction. (d) XPS survey spectra of bare-Al_2_O_3_ and DA + PSBMA-Al_2_O_3_. (e) Corresponding high-resolution XPS spectra in the N 1s region. (f) Deposition time dependence of contact angles for DA + PSBMA-Al_2_O_3_.

Before implementing surface modification of the nanoporous AAO membrane through the proposed codeposition of DA and PSBMA, we utilized a flat aluminum oxide substrate, denoted as Al_2_O_3_, to demonstrate successful surface modification through DA-assisted codeposition for 24 h using PSBMA with an *M_n_* of 18.4 kg/mol. By observing the water contact angles on bare Al_2_O_3_ (bare Al_2_O_3_: 12.4°) and the modified Al_2_O_3_ (DA + PSBMA-Al_2_O_3_: ∼0°), the increasing hydrophilicity of the DA + PSBMA-Al_2_O_3_ implies the successful formation of PSBMA coatings on the Al_2_O_3_ surface (Figure 2a). To validate the function of DA as effective linkers between PSBMA and Al_2_O_3_ substrates, contact angles of the coatings prepared by immersion in pure PSBMA and pure DA solutions were also recorded (Figure S2). Compared to the bare Al_2_O_3_ (12.4°), the DA-Al_2_O_3_ shows a relatively more hydrophobic surface with an increased contact angle of 39.5°, while the PSBMA-Al_2_O_3_ shows similar hydrophilicity with a nearly unchanged contact angle of 15°. This indirectly validates that the DA moiety must interact with both the PSBMA and Al_2_O_3_ surfaces simultaneously to achieve the increased hydrophilicity observed in the DA + PSBMA-Al_2_O_3_ system (Figure 2b). In the ultraviolet–visible (UV–vis) spectra of the DA and DA + PSBMA solutions after a 4 h incubation under an air atmosphere (Figure 2c), a red-shifted absorption peak at 305 nm in the DA + PSBMA solution further implies the delocalization of the π-electron in dopamine with PSBMA through the formation of a cation−π interaction. The chemical composition of the coating on the DA + PSBMA-Al_2_O_3_ was further identified by X-ray photoelectron spectroscopy (XPS). In the XPS survey spectra (Figure 2d), the presence of N 1s and S 2s peaks, as well as the absence of Al 2s and Al 2p peaks for DA + PSBMA-Al_2_O_3_, confirms that the formed coating contains the hydrophilic PSBMA moiety and covers the Al_2_O_3_ substrate well, respectively. The high-resolution XPS spectra of N 1s for DA + PSBMA-Al_2_O_3_ show two peaks at ∼401 and ∼399 eV (Figure 2e), associated with the quaternary ammonium (C–N^+^) on the zwitterionic PSBMA moiety and amine (C–N) on the self-polymerized DA moiety, respectively.^31^ This verifies that the DA can robustly immobilize PSBMA through specific interactions with the sulfobetaine moiety and anchor it to the alumina surfaces, providing a possible route for the surface modification of porous AAO membranes. The theoretical and measured values of the atomic ratio among C 1s, O 1s, N 1s, S 2p, and Al 2p are summarized in Table 1. The DA + PSBMA coating coverage (CC) on the Al_2_O_3_ surfaces can be evaluated from the ratio of Al 2p/O 1s. Both high CC values (>95%) of the DA-Al_2_O_3_ and the DA + PSBMA-Al_2_O_3_ reveal excellent biomimetic adsorption of the DA moiety for simple coating preparation on Al_2_O_3_ substrates. The theoretical molar ratio (MR) of SBMA to DA units (SBMA/DA) is the same as the feeding ratio, which is 3.39:1, while the molar ratio of SBMA/DA for DA + PSBMA-Al_2_O_3_ is measured to be ∼0.44:1 from the S 2p/N 1s ratio, assuming that surface composition of the deposited coating is in accordance with that of the inner layer. The fraction of PSBMA in the aggregated particles of the DA + PSBMA coating is much lower than the feeding ratio, consistent with the observation in the previous report.^32^ This finding suggests that only a few PSBMA chains interacted with self-polymerized DA to form DA + PSBMA coatings and codeposited on the surfaces for improving the surface hydrophilicity, while most of the water-soluble PSBMA chains acted like surfactants, surrounding the water-insoluble and relatively hydrophobic DA + PSBMA aggregated particles in the solution, and were removed from the coating surfaces after ultrasonication washing. To determine the optimal codeposition time period, we conducted water contact angle measurements (Figure S3) and XPS analysis (Figure S4) on the DA + PSBMA-Al_2_O_3_ samples prepared with various time periods. The results show that longer codeposition times lead to lower contact angles (Figure 2f), indicating a more hydrophilic surface due to the incorporation of the PSBMA moiety and the absence of an Al element signal (Table S1), indicating almost full coverage. Consequently, we performed subsequent surface modifications on all the nanoporous AAO membranes with a fixed codeposition time of 24 h.

**Table 1:** XPS Results of DA + PSBMA Coatings on Al_2_O_3_ and AAO Substrates

		atomic ratio^a^ (%)								
sample		C 1s	O 1s	N 1s	S 2p	Al 2p	S 2p /N 1s	MR^b^ (mol/mol)	Al 2p /O 1s	CC^c^ (%)
theoretical value	PSBMA	61.1	27.8	5.5	5.5	0	1			
	DA	72.7	18.2	9.1	0	0	0			
	DA + PSBMA	63.7	25.6	6.3	4.2	0	0.67	3.39:1		
on the flat substrate	bare-Al_2_O_3_	14.4	62.4	0.9	<0.1	22.3				
	DA-Al_2_O_3_	65.7	25.8	8.4	<0.1	0.2	<0.1	0.00:1	0.008	>98
	DA + PSBMA-Al_2_O_3_	64.1	26.5	7.7	1.8	<0.1	0.23	0.44:1	0.004	>99
in the AAO nanopore	bare-AAO	28.5	53.6	0.6	1.3	16.0				
	DA + PSBMA-AAO	63.0	27.2	6.5	2.5	0.8	0.38	0.86:1	0.029	95.6

a The atomic ratio is obtained by integrating the peak area in the survey XPS spectra, divided by the sensitivity factor of the corresponding element.

b The molar ratio (MR) of SBMA/DA on the surface. The MR value can be derived from S 2p /N 1s by assuming that the surface composition of the deposited coating is in accordance with that of the inner layer.

c Coating coverage (CC) on the Al_2_O_3_ surfaces. The CC value was calculated by [1–1.5(Al 2p/O 1s)] × 100 (%).

### Surface Modification on the Porous AAO Membrane through DA + PSBMA Codeposition

**Figure 3:**
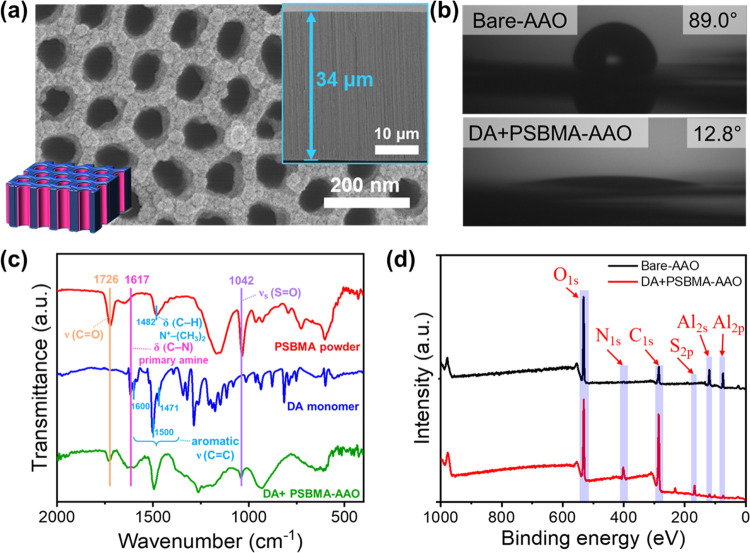
Characterizations of the DA + PSBMA-AAO membrane. (a) Schematic illustration and top-view SEM image of the DA + PSBMA-AAO membrane, revealing that aggregated PDA/PSBMA nanoparticles deposited on the AAO membrane with a pore diameter of ∼73 nm. The inset is the cross-sectional SEM image of the DA + PSBMA-AAO membrane, showing a membrane thickness of ∼34 μm. (b) Water contact angles on bare-AAO (∼89°) and DA + PSBMA-AAO (∼13°). The increasing hydrophilicity implies the successful codeposition on the AAO membrane surfaces. (c) FTIR spectra of PSBMA powder, DA monomer, and DA + PSBMA-AAO membrane. (d) XPS analysis on the cross-sections of bare-AAO and DA + PSBMA-AAO.

A feasible surface modification route through the codeposition of DA and PSBMA was demonstrated on a nanoporous AAO membrane. The morphology of the modified DA + PSBMA-AAO membrane was observed using scanning electron microscopy (SEM). Compared to the SEM images of the bare-AAO membrane (Figure S5), SEM images (Figure 3a) clearly show aggregated DA + PSBMA coatings on the top surface of the DA + PSBMA-AAO membrane with a pore size of ∼73 nm and thickness of ∼34 μm. This indicates the successful application of the proposed codeposition method to the porous substrates. Similarly, the improved hydrophilicity due to the presence of the PSBMA moiety can be observed in the contact angle results (Figure 3b). The chemical composition of the coated layer on the top surface of the DA + PSBMA-AAO membrane was identified using Fourier–transform infrared (FTIR) spectroscopy combined with an attenuated total reflectance (ATR) method (Figure 3c). On the one hand, when comparing the FTIR spectrum of PSBMA powder, the spectrum of the DA + PSBMA-AAO membrane exhibits two characteristic peaks: the carbonyl group (C=O) stretching at 1726 cm^–1^ and sulfonate (S=O) asymmetric stretching at 1042 cm^–1^.^38,39^ These peaks confirm once again that the aggregated coatings on the AAO top surfaces contain PSBMA, which is immobilized by the DA moiety through possible interactions such as ammonium cation–π interactions, hydrogen bonding, and chain entanglement. On the other hand, the two broader peaks at ∼1617 and ∼1500 cm^–1^ in the spectrum of the DA + PSBMA-AAO are associated with a combination of peaks involving the bending of C–N in primary amine and C–N^+^ in quaternary ammonium and the stretching of C=C in the aromatic ring.^40,41^ This verifies that the coating on the AAO top surface is composed of both PSBMA and DA.

To assess the efficacy of codeposition in modifying the cylindrical surfaces within the nanopores of the AAO membrane, we employed a focused X-ray with a beam size of ∼10 μm and a specialized cross-sectional holder to conduct elemental analysis on the cross-section of the DA + PSBMA-AAO membrane using XPS (Figure S6). Similar to the XPS results obtained from the flat Al_2_O_3_ substrates (Figure 2d), the XPS results from the cross-sectional surfaces of the AAO membranes indicate the presence of N 1s and S 2p peaks, along with a reduction of Al 2s and Al 2p peaks after the codeposition process (Figure 3d). Further evidence of the noticeable N and S signals can be observed through the high-magnification mapping SEM images coupled with energy-dispersive X-ray (EDX) spectroscopy in a selected region on the cross-sectional surface of the DA + PSBMA-AAO membrane (Figure S7). These findings strongly validate the capability of the codeposition method for surface modification on nanoconfined surfaces. The high-resolution cross-sectional XPS spectra of N 1s (Figure S8) also display two peaks associated with the quaternary ammonium (C–N^+^) and amine (C–N), indicating that the surface coatings within the AAO nanopores consist of both PSBMA and self-polymerized DA. The atomic ratio obtained from the XPS analysis on the cross-sectional surface of the DA + PSBMA-AAO membrane is also summarized in Table 1. The higher SBMA/DA molar ratio (∼0.86:1) for the DA + PSBMA-AAO membrane, compared to that (∼0.44:1) for the DA + PSBMA-Al_2_O_3_, suggests that the confinement of the AAO nanopores may lead to a more significant fraction of PSBMA immobilized by the DA component in the aggregated coatings.

### DA + PSBMA Coatings in Nanoscale Confinement: Effects of the Molecular Weight of PSBMA and Pore Size of AAO

**Figure 4:**
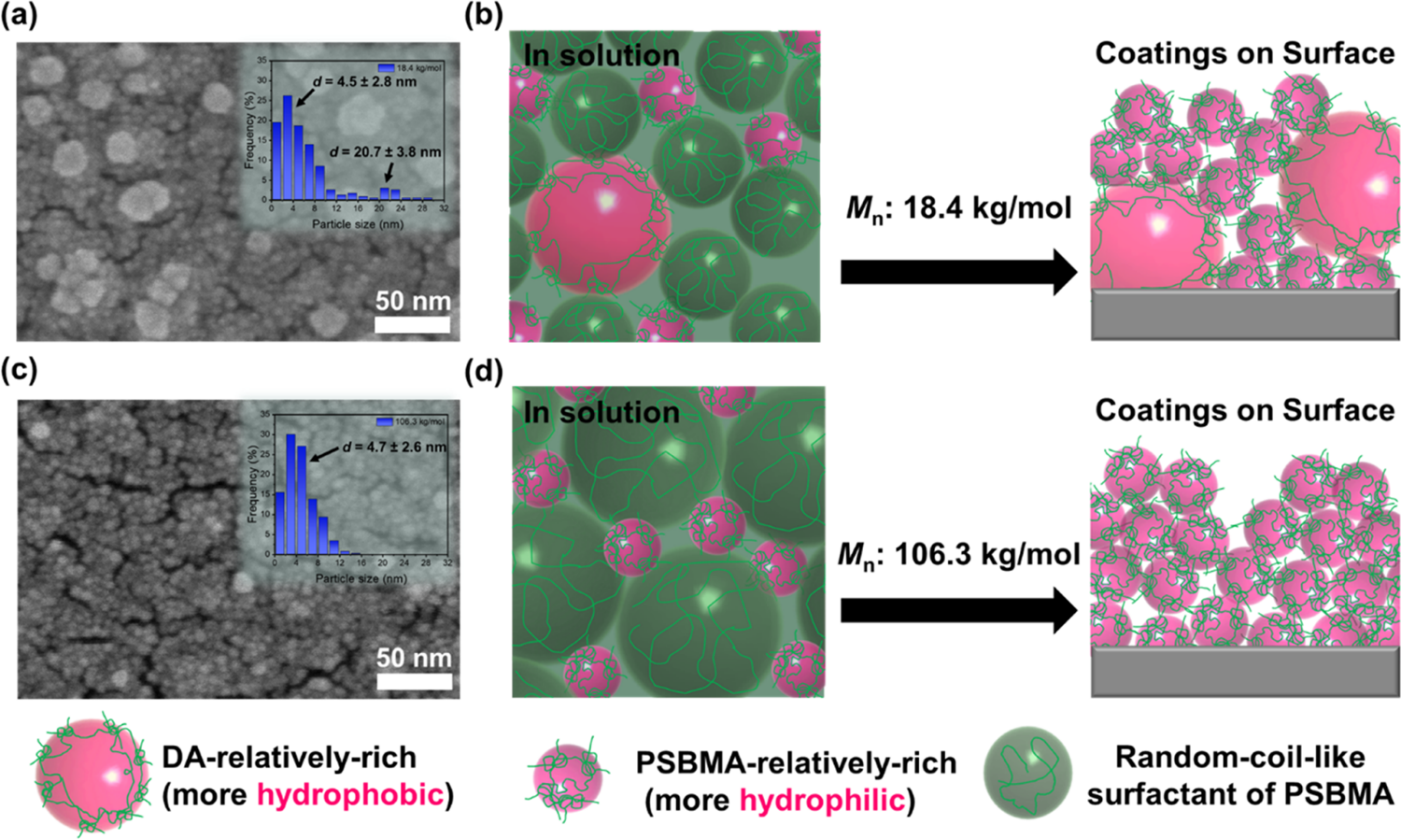
SEM results and proposed formation mechanism of DA + PSBMA aggregated nanoparticles in solutions and coatings on glass surfaces using PSBMA with molecular weights (*M_n_*) of (a–b) 18.4 kg/mol and (c–d) 106.3 kg/mol after a 24 h codeposition time period.

To gain deeper insights into the confinement effect on the aggregated DA + PSBMA nanoparticle coatings in nanopores, control group experiments with no confinement were first carried out by codepositing freely grown DA + PSBMA nanoparticles on flat glass substrates. When the same molecular weight (*M_n_*: 18.4 kg/mol) of PSBMA that was used in the AAO nanopore was also applied on the flat surface, two apparent particle sizes of 4.5 ± 2.8 nm and 20.7 ± 3.8 nm were observed in the SEM image, indicating a broader and bimodal particle size distribution (Figure 4a). These larger aggregated particles suggest the presence of free PDA with little/no incorporation of hydrophilic polyzwitterions^30,42^ and may be inhibited from growing inside the AAO nanopores due to the crowded nanoscale confinement, thus increasing the SBMA/DA molar ratio to 0.86 shown in Table 1. A possible mechanism of the DA + PSBMA nanoparticle formation in solutions and the corresponding coatings deposited on the flat surface is proposed in Figure 4b. In the solution, as the DA starts to self-polymerize and grow into water-insoluble aggregated nanoparticles, a few PSBMA chains at interfaces entangle and/or interact with the self-polymerized DA moiety to form more hydrophilic DA + PSBMA nanoparticles, while most of the water-soluble PSBMA chains, still dissolving in the aqueous solution, may form random coil-like polymeric surfactants to inhibit further coalescence. When using a smaller *M_n_* of 18.4 kg/mol, the random coil-like PSBMA surfactants with a smaller root-mean-square radius of gyration (*R*_g_) of ∼2.8 nm (estimated by *R*_g_ =  × 2.44 Å based on the freely jointed chain model using a characteristic ratio of 15.49)^43^ may have faster Brownian motion in the solution, allowing oxygen from the air to transfer more easily into the water-insoluble domain for further self-polymerization into larger and relatively DA-rich DA + PSBMA nanoparticles. Consequently, DA + PSBMA coatings with broader and bimodal particle size distribution were deposited on the flat glass surface. As the *M_n_* increases to 106.3 kg/mol, the formation of the relatively larger particles is significantly mitigated, and a single-size particle distribution for the relatively smaller particles (4.7 ± 2.6 nm) becomes evident (Figure 4c). The corresponding calculated *R*_g_ of the random coil-like PSBMA surfactants will increase to ∼6.7 nm,^43^ and the decreased Brownian motion in the solution may prevent oxygen from transferring into the water-insoluble domain, contributing to a more homogeneous coating morphology on the surface with a single-size particle distribution (Figure 4d). Since the SBMA/DA feeding ratio (3.39:1) and PSBMA concentration (10 mg/mL) were fixed in both lower and higher *M_n_* cases, the mitigated formation of the larger and relatively DA-rich aggregation through the use of higher *M_n_* is highly relevant due to the enhanced separating ability of the longer chain PSBMA surfactant. These findings can be further supported by the XPS results measured on the DA + PSBMA-coated glass surfaces (Figure S9 and Table S2), which show a higher SBMA/DA molar ratio (MR = 1.64) for the sample using an *M_n_* of 106.3 kg/mol compared to that of 18.4 kg/mol (MR = 0.25).

**Figure 5:**
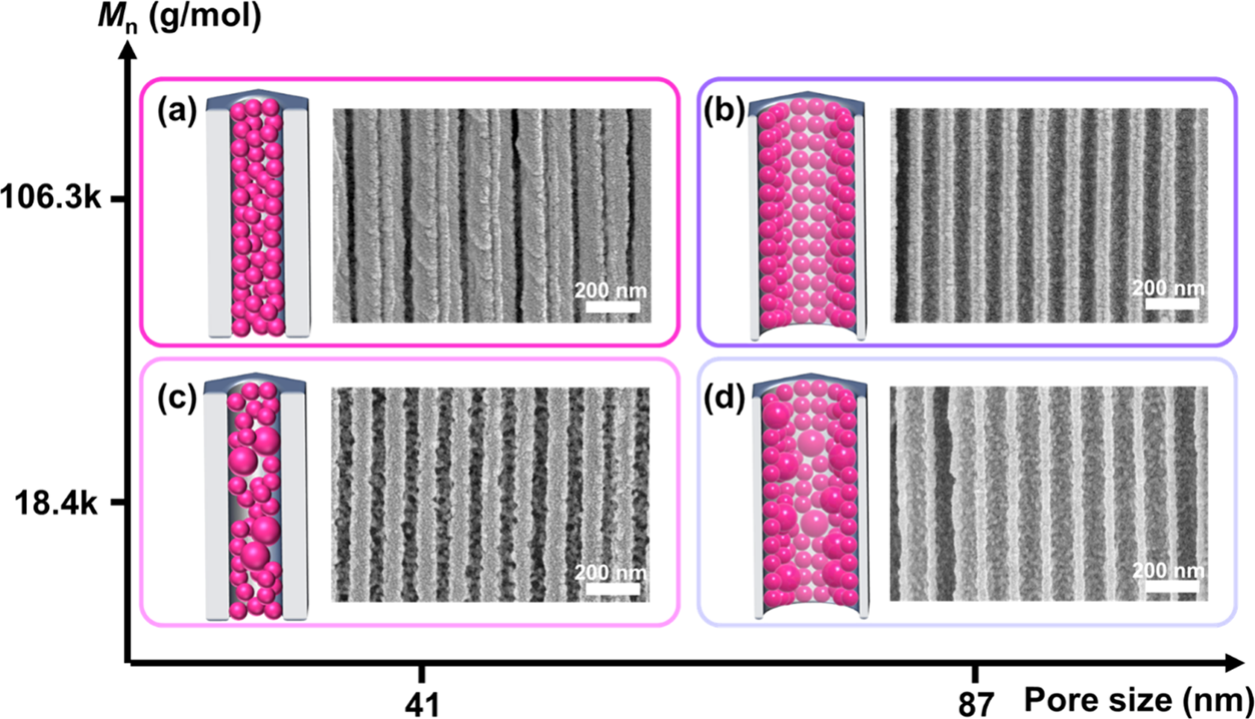
Effects of PSBMA molecular weights (*M_n_*) and AAO pore sizes on codeposited coating morphologies within nanospaces. Schematic illustrations and cross-sectional SEM images of DA + PSBMA-AAO membranes fabricated with varying *M_n_* and pore sizes of (a) 106.3 kg/mol and 41 nm, (b) 106.3 kg/mol and 87 nm, (c) 18.4 kg/mol and 41 nm, and (d) 18.4 kg/mol and 87 nm.

To investigate the codeposition within confined nanoscale spaces, we used AAO membranes with varying pore sizes (∼41 and ∼87 nm) and PSBMA with different molecular weights (*M_n_*: 18.4 and 106.3 kg/mol) during the surface modification (Figures 5 and S10). When smaller pore sizes (41 nm) were employed, the DA + PSBMA nanoparticles filled most of the spaces within the AAO nanopores, creating pseudo-rod-like nanostructures, resembling nanorods with defects/cavities, within the nanopores (Figure 4a,c). In contrast, with larger pore sizes (87 nm), the DA + PSBMA nanoparticles primarily occupied the spaces near the pore walls, forming hollow tube-like nanostructures, essentially cylindrical thin film coatings, within the nanopores (Figure 4b,d). Our experimental investigation on the effect of pore size reveals that varying the pore size can significantly alter the extent of pore filling and the morphology of the DA + PSBMA nanoparticle deposition within the nanopores. Furthermore, when a higher *M_n_* (106.3 kg/mol) was used, the formed DA + PSBMA nanostructures exhibited fewer defects/cavities due to the closer packing of single-sized aggregated nanoparticles on the confined walls, resulting in a much more homogeneous membrane modification within the nanopores, as illustrated in the cartoons in Figure 5a,5b. On the other hand, with lower *M_n_* (18.4 kg/mol), the resulting DA + PSBMA nanostructures exhibited more defects/cavities due to the less efficient packing of two-sized aggregated nanoparticles, leading to a less homogeneous membrane modification within the nanopores, as illustrated in the cartoons in Figure 5c,5d. These findings regarding the effect of *M_n_* suggest that using polyzwitterions with high molecular weights or longer chain lengths is recommended for achieving homogeneous membrane modification through DA-assisted codeposition within the nanospaces.

### Stability of DA + PSBMA-Codeposited Coatings on the AAO Membrane

The stability of the DA + PSBMA-codeposited coatings was evaluated both under an air atmosphere and in pure water medium. The coating stability under an air atmosphere was investigated by XPS analysis using the DA + PSBMA-Al_2_O_3_ stored in ambient conditions for a longer period, up to 8 months (Figure S11). The corresponding molar ratio of SBMA/DA and coating coverage are summarized in Table S3, showing excellent stability in the air with high coating coverage (over 99%) and little change in the SBMA/DA molar ratio after 8 months.

**Figure 6:**
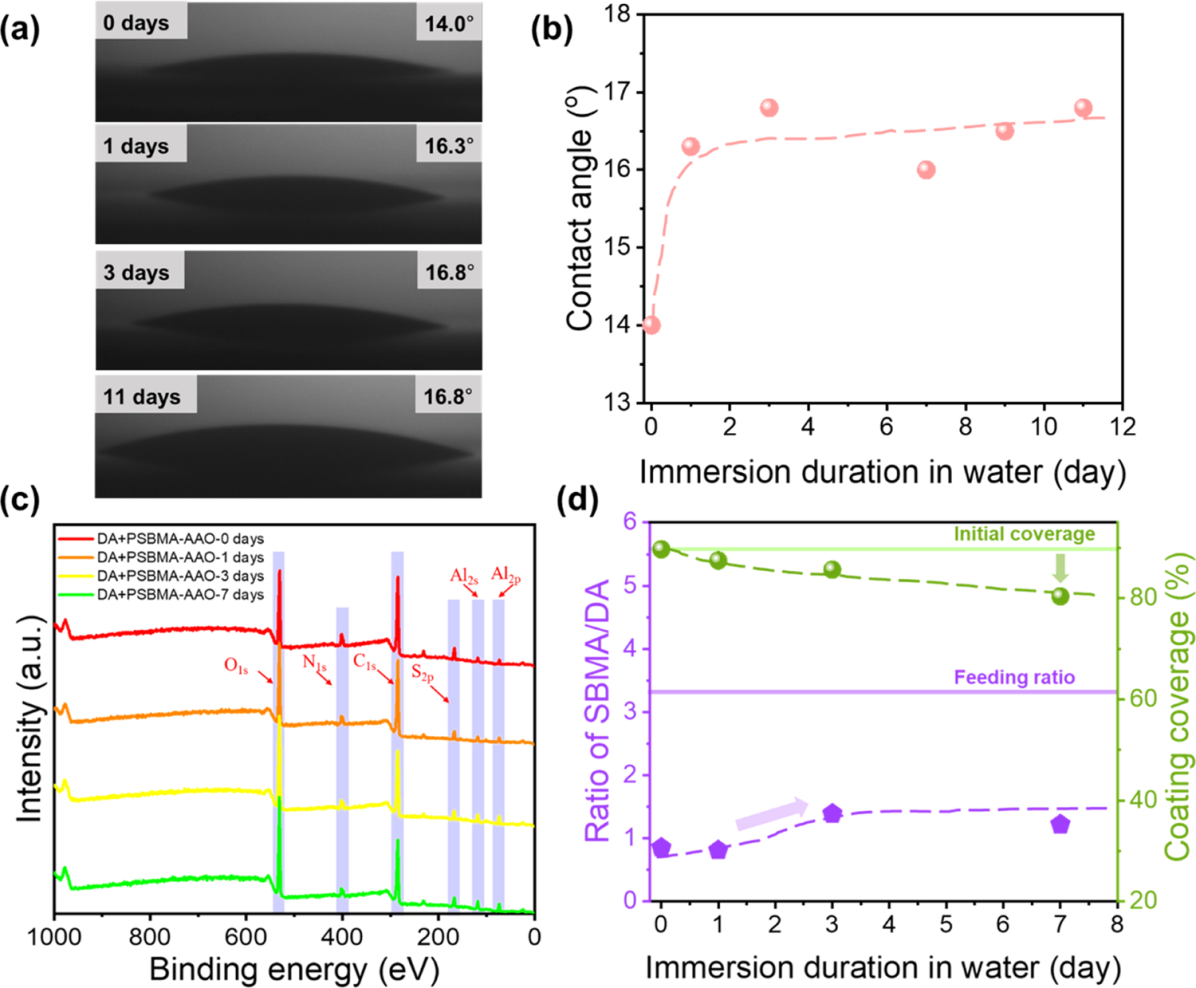
Stability test of DA + PSBMA-AAO (*M_n_* of PSBMA: 18.4 kg/mol) under different immersion durations in water. (a) Water contact angles on DA + PSBMA-AAO after immersion in water for 0, 1, 3, and 11 days. (b) Time dependence of water contact angles for DA + PSBMA-AAO stored in pure water media. (c) XPS survey spectra on top surfaces of DA + PSBMA-AAO after immersion in water for 0, 1, 3, and 7 days. (d) Time dependence of the SBMA/DA molar ratio and coating coverage for DA + PSBMA-AAO stored in pure water media.

**Figure 7:**
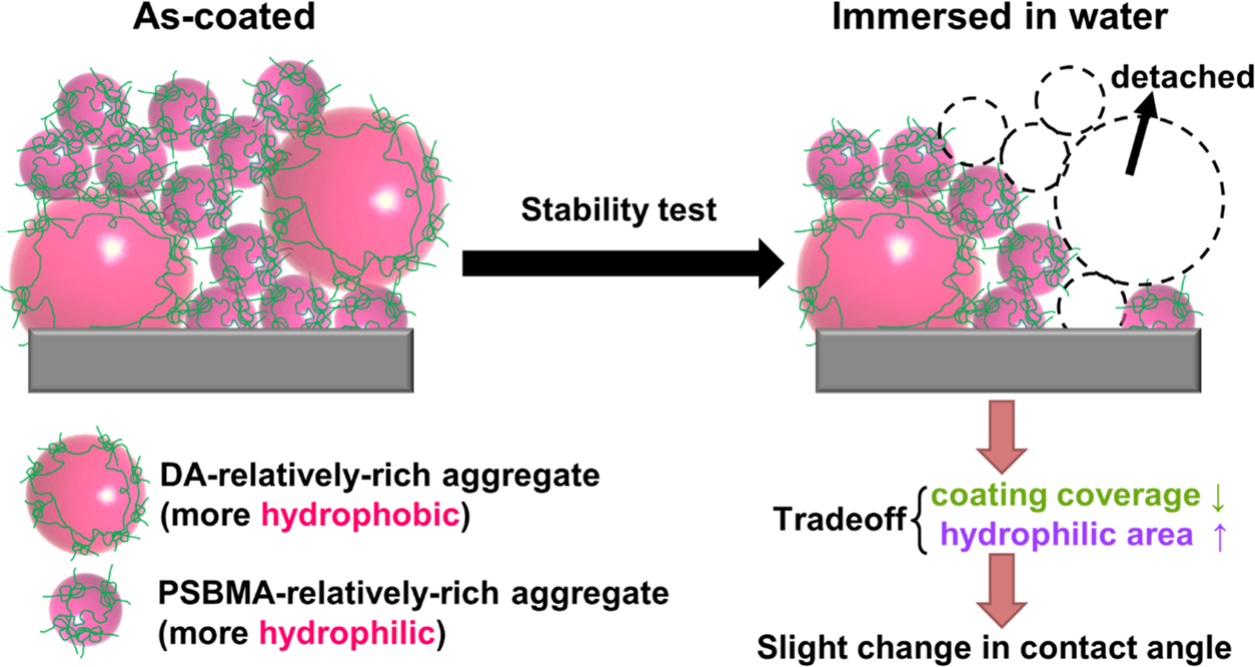
Illustrative scheme depicting the slight change in the water contact angle after long-term water immersion, showing a trade-off between the decrease in coating coverage and the increase in hydrophilic surface areas after the detachment of the larger and more hydrophobic DA-relatively rich aggregated DA + PSBMA nanoparticles.

The stability of the coating in pure water media was investigated through water contact angle measurement and XPS analysis using the dried DA + PSBMA-AAO immersed in the water media for various durations (Figure 6). The water contact angle results (Figure 6a–b) showed only a slight change (<3°) in the wettability of the DA + PSBMA-AAO, indicating satisfactory stability performance in surface hydrophilicity. To further understand the correlation between wettability and nano/molecular-scale changes at the interfaces on the top surface of the AAO membrane during the stability test in pure water media, surface composition changes after different water immersion durations were recorded by XPS analysis and summarized (Figure 6c and Table 2). After a 7-day water immersion, the coating coverage slightly decreases by ∼10%, while the molar ratio of SBMA/DA, surprisingly, increases by ∼50% (Figure 6d). The decrease in coating coverage is possibly associated with the detachment of the relatively hydrophobic and larger DA + PSBMA nanoparticles on the top surfaces of AAO membranes, resulting in a negative impact on the surface hydrophilicity of the membrane. Notably, the increase in the molar ratio of SBMA/DA after the detachment of DA-relatively rich nanoparticles is reasonably expected to have a positive impact because more hydrophilic and PSBMA-relatively rich nanoparticles left more area on the top surfaces of the membrane. The satisfactory stability performance of the AAO surfaces can be considered a trade-off outcome between the decrease in coating coverage and the increase in hydrophilic areas after the water immersion, as illustrated in the scheme shown in Figure 7.

**Table 2:** XPS Results of DA + PSBMA-AAO after Different Immersion Durations in Water

	atomic ratio^a^ (%)								
immersion duration in water	C 1s	O 1s	N 1s	S 2p	Al 2p	S 2p /N 1s	MR^b^ (mol/mol)	Al 2p /O 1s	CC^c^ (%)
0 day	63.4	24.7	7.4	2.8	1.7	0.38	0.85	0.07	89.7
1 day	62.7	26.5	6.3	2.3	2.2	0.37	0.81	0.08	87.5
3 days	58.4	30.3	5.2	2.6	3.5	0.50	1.39	0.12	82.7
7 days	55.7	31.4	5.9	2.8	4.1	0.47	1.22	0.13	80.4

a The atomic ratio is obtained by integrating the peak area in the survey XPS spectra, divided by the sensitivity factor of the corresponding element.

b The molar ratio (MR) of SBMA/DA on the surface.

c The coating coverage (CC) value was calculated by [1–1.5(Al 2p /O 1s)] × 100 (%).

Although the stability properties in hydrophilicity on the top surface of the membrane may still differ from those inside the nanopores, this work provides some clues to suggest using a higher *M_n_* of polyelectrolyte during codeposition for improving the coating homogeneity and reducing coating detachment in water media. In the future, we will continue our efforts to gain a deep understanding of membrane hydrophilicity at the single-pore scale, such as by investigating capillary water imbibition in AAO membranes^44^ and aim to demonstrate its applicability by testing the membrane’s performance in oil/water separation and ion screening.

## Conclusions

This study has successfully demonstrated a straightforward method for the surface modification of nanoporous AAO membranes using dopamine-assisted codeposition with PSBMA polymers. The codeposition process effectively formed aggregated coatings of DA and PSBMA nanocomposites within the nanoporous AAO membranes. The investigation also explores the influence of various factors, including PSBMA molecular weights and AAO pore sizes, on the morphology and uniformity of the nanocomposite coatings within the nanopores. It was observed that higher molecular weights of PSBMA favor more uniform modifications, suggesting better stability of hydrophilicity performance in water media. This research advances our understanding of membrane modification techniques for nanoscale environments achieved through functionalization based on the codeposition of DA and zwitterionic polymers under ambient conditions. It paves the way for the development of simple and efficient membrane modification processes, offering a promising avenue for broader utilization.

## Experimental Section

### Materials

All the following chemicals were used without further purification: sulfobetaine methacrylate (SBMA, 98%, Taiwan Hopax Chemicals), 4,4′-Azobis(4-cyanovaleric acid) (ACVA, 98%, Sigma-Aldrich), 4-cyanopentanoic acid dithiobenzoate (CPAD, 97%, Strem Chemicals), dopamine hydrochloride (DA, 98%, Tokyo Chemical Industry), ethanol (95%, ECHO), acetone (99%, ECHO), isopropyl alcohol (IPA, 95%, Uni-Onward), methanol (95%, Uni-Onward), deuterated water (D_2_O, 99.9%, Sigma-Aldrich), 2,2,2-trifluoroethanol (TFE, 99.8%, Acros Organics), oxalic acid (H_2_C_2_O_4_, 97%, Showa), chromium(VI) oxide (CrO_3_, 98%, Showa), copper chloride (CuCl_2_, 98% Showa), hydrochloric acid (HCl, >37%, Honeywell Fluka), phosphoric acid (H_3_PO_4_, >85% J. T. Baker), and perchloric acid (HClO_4_, 70%, Showa). Deionized (DI) water was obtained by a pure water system (RODA) with a resistivity of 18.25 MΩ·cm. A 0.1 M tris(hydroxymethyl)aminomethane (Tris) buffer solution with pH = 8.1 was prepared by dissolving a preset pHast Pack powder (PPB023, Sigma-Aldrich) in DI water to a total volume of 500 mL. 0.2 mm-thick aluminum foil (Al foil, 99.997%), 1 mm-thick aluminum oxide (Al_2_O_3_), and 1 mm-thick glass substrates were procured from Alfa Aesar, Cheng Yang Instrument Corp., and FEA, respectively.

### Synthesis and Characterization of PSBMA

PSBMA was synthesized with controlled molecular weight and molecular weight distribution via RAFT polymerization. In the general procedure, SBMA (6.0 g, 21.000 mmol) as the monomer, ACVA (11.8 mg, 0.042 mmol) as the initiator, and CPAD (58.7 mg, 0.210 mmol) as the chain transfer agent (CTA) were dissolved in a 21 mL mixed solution of water and methanol (v/v = 1:1) in a round-bottom flask. The solution was then degassed using nitrogen bubbling for 20 min. Subsequently, the degassed solution was then heated to 55 °C for 23 h to polymerize the PSBMA. To prepare PSBMA with different molecular weights, varying expected degrees of polymerization (calculated from the [SBMA]_0_/[CPAD]_0_), such as 100, 500, and 1000, were employed during the RAFT polymerization with a fixed CPAD/AVCA ratio of 5. The chemical structures of the synthesized PSBMA were characterized using ^1^H NMR, FTIR spectroscopy, and TGA. The NMR spectrum was acquired using a 600 MHz NMR spectrometer (Agilent) with a PSBMA solution in D_2_O. The FTIR spectrum of PSBMA powder was measured in the range of 650–4000 cm^–1^ with a resolution of 4 cm^–1^, accumulating 64 scans using an FT/IR-4X spectrometer (Jasco) equipped with an ATR accessory with a diamond crystal using the air as the reference. The TGA thermogram was obtained by combusting 8.84 mg of PSBMA powder in the 25–550 °C temperature range using a TGA2950 thermogravimetric analyzer (TA Instrument) with a heating rate of 10 °C/min. The molecular weight and molecular weight distribution of the synthesized PSBMA were evaluated using an HLC-8320 gel permeation chromatography (GPC, Tosoh) system connected with a guard TSKgel SuperAW-H column (Tosoh) and 3 SuperAWM-H columns (Tosoh) at 40 °C with a flow rate of 0.6 mL/min and TFE as the eluent.

### Fabrication, Surface Modification, and Characterization of DA + PSBMA-AAO Membranes

The AAO membranes were fabricated using a modified two-step anodization process.^45−47^ In the typical procedure, the high-purity Al foil was cut into several pieces (12 mm × 50 mm) and then washed in sequence with acetone, IPA, and water using ultrasonication for 10 min during each step. Subsequently, an electropolishing process was performed using an HClO_4_/methanol (v/v = 1:4) solution. The polished Al foils were anodized using 0.3 M H_2_C_2_O_4_(aq) at 50 V for 30 min at 20 °C. The formed oxide layer was then removed through chemical etching using a mixed solution of CrO_3_/H_3_PO_4_ (3.6:6.0 wt %). The second anodization was conducted for 2 h under the same conditions as the first anodization. The pore sizes could be further controlled through a pore-widening process using 5 wt % H_3_PO_4_(aq) for 0, 30, and 50 min.

The surface modification of the prepared AAO membrane was conducted through the codeposition of PSBMA and DA in a Tris buffer solution. In the general procedure, PSBMA (100 mg) and DA (20 mg), with this fixed weight ratio, were dissolved in 10 mL of the as-prepared 0.1 M Tris buffer solution (pH 8.1). Subsequently, the AAO membranes were immersed in the fresh DA + PSBMA solution for codeposition with various time periods on a TS-520D shaker (YIHDER) stage at a shaking rate of 60 rpm. After deposition, the resulting DA + PSBMA-AAO membrane was washed with DI water using ultrasonication for 2 min to remove the nonadsorbed PSBMA, DA, and PDA molecules. The membranes were then dried and stored under reduced pressure conditions before further characterization. The surfaces of the flat Al_2_O_3_ and glass substrates were also modified using the same codeposition process for comparison.

The surface hydrophilicity of the modified AAO membranes was assessed through static contact angle measurements using an FTA125 goniometer system (KSV NIMA Instruments) with 3 μL of DI water as the probe liquid. All photos shown in the contact angle measurements were captured once the water droplet fully detached from the needle and just fell onto the top surfaces of the samples, and the contact angles were determined by averaging values obtained from at least three tests. The electron transition properties for diluted DA (0.5 mg/mL) and DA + PSBMA (0.5 + 2.5 mg/mL) solutions after a 4 h incubation in the air were investigated using a DH-2000-BAL UV–vis spectrometer system (Ocean Optics). The chemical structures of coatings on the surfaces of the modified AAO membranes were identified using the aforementioned FT/IR-4X spectrometer with the same parameters. The morphologies of top and cross-sectional surfaces of both the as-fabricated and modified AAO membranes were characterized under an accelerating voltage of 3 kV using an S-4800 SEM (Hitachi) equipped with an XFlash 6–30 EDX detector (Bruker). For SEM measurements, all samples were coated with a thin layer of Pt using an E-1010 ion sputter (Hitachi) for 60 s to enhance the conductivity. Elemental analysis of the cross-sectional AAO surfaces was also conducted using a Versa Probe 4 X-ray photoelectron spectrometer (ULVAC PHI) under a vacuum of 2 × 10^–9^ Torr with a monochromatic Al–Kα X-ray source (1486.6 eV). The cross-sectional XPS spectra were obtained by fixing the AAO membranes on a customized cross-sectional holder and focusing the X-ray beam (beam size: ∼10 μm) on the sample with the assistance of microscopy.

## Supporting Information Available

^1^H NMR spectra, TGA thermogram, GPC traces, photos and contact angle images of time-dependent codeposition, cross-sectional XPS holder images, XPS spectra, and SEM/EDX mapping images of AAO membranes and DA+PSBMA nanoparticles (PDF)
