# Supplementary material for: Surface Modification of Nanopores in an Anodic Aluminum Oxide Membrane through Dopamine-Assisted Codeposition with a Zwitterionic Polymer
Source: Langmuir. 2024 Feb 26;40(10):5245–54. doi: 10.1021/acs.langmuir.3c03654 (PMC10938887; doi:10.1021/acs.langmuir.3c03654)
Supplement: Supplementary file 1 — la3c03654_si_001.pdf [file la3c03654_si_001.pdf]

Supporting Information

Surface Modification of Nanopores in Anodic Aluminum Oxide Membrane through Dopamine-Assisted Co-Deposition with Zwitterionic Polymer

Chien-Wei Chu [a*], *Chia-Hsuan Tsai* [a]

[a] *Department of Chemical Engineering, Feng Chia University, Xitun District, Taichung City, 40724, Taiwan.*

*Email: cwchu@fcu.edu.tw

Synthetic route and characterizations of the PSBMA via RAFT polymerization

Labeling of the synthesized PSBMA in ^{1}H-NMR in D_2O (Figure S1b): δ_H 0.00−1.62 (3H, H_a), 1.62−2.19 (3H, H_b), 4.46 (2H, H_c), 3.87 (2H, H_d), 3.30 (6H, H_e), 3.65 (2H, H_f), 2.34 (2H, H_g), 3.04 (2H, H_h) on polymer chains and δ_H 7.62 (2H, H_i), 7.79 (2H, m, $H_{j,}$) 8.05 (1H, H_k) in the aromatic ring on the RAFT agent. In TGA results (Figure S1c), the initial weight loss at ~300 °C corresponds to the structural changes in both the backbones and pendant groups of PSBMA chains, and the subsequent weight loss at ~420 °C can be attributed to the degradation of ammonium and sulfonate groups.

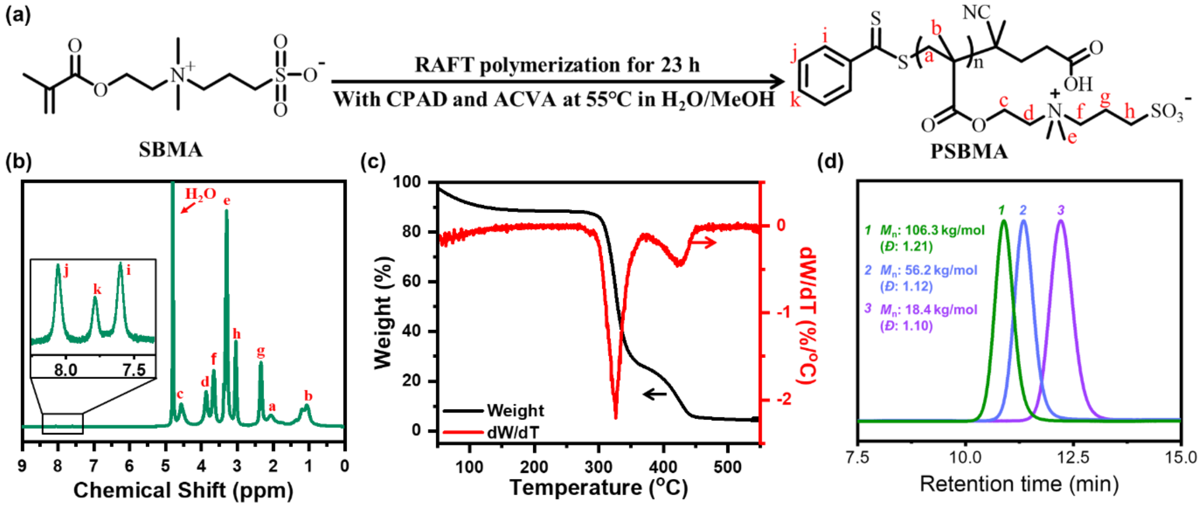

Figure S1. Synthesis and Characterizations of PSBMA: (a) Synthetic route using RAFT polymerization, (b) ^{1}H NMR spectrum, (c) TGA thermogram, and (d) GPC trace of the synthesized PSBMA.

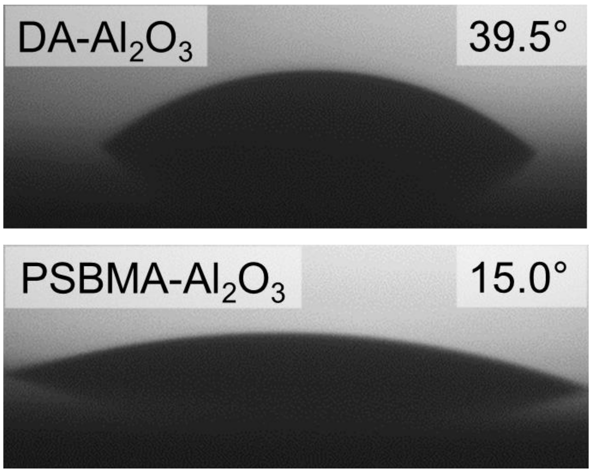

Figure S2. Water contact angles on the DA-coated substrate (DA-Al_2O_3: 12.4°) and the PSBMA-coated substrate (PSBMA-Al_2O_3: 15°) after ultrasonication washing. The DA-Al_2O_3 shows a relatively more hydrophobic surface with an increased contact angle of 39.5°, while the PSBMA-Al_2O_3 shows similar hydrophilicity with a nearly unchanged contact angle of 15°.

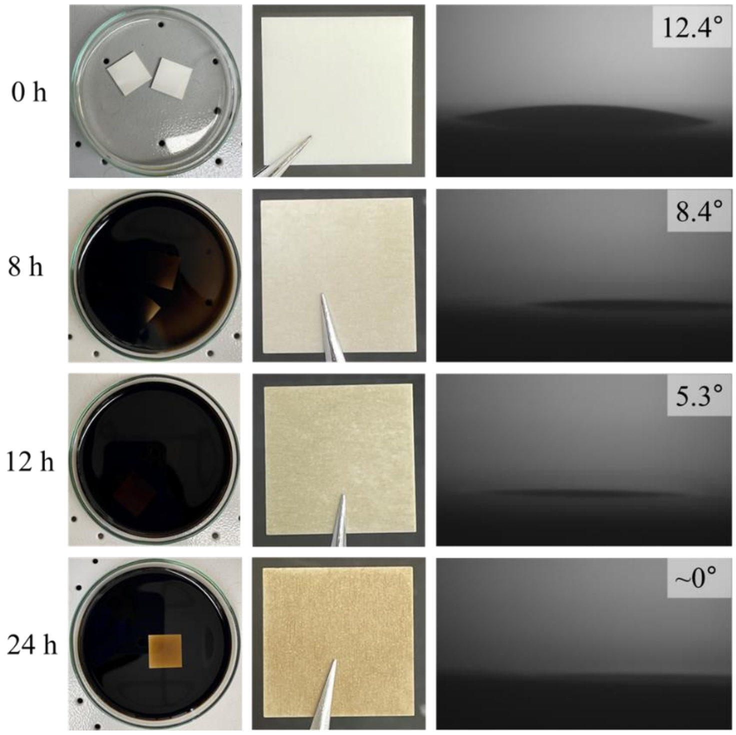

Figure S3. Photos and contact angle results for surface modification on flat Al_2O_3 substrates through co-deposition of DA and PSBMA for 0, 8, 12, and 24 h.

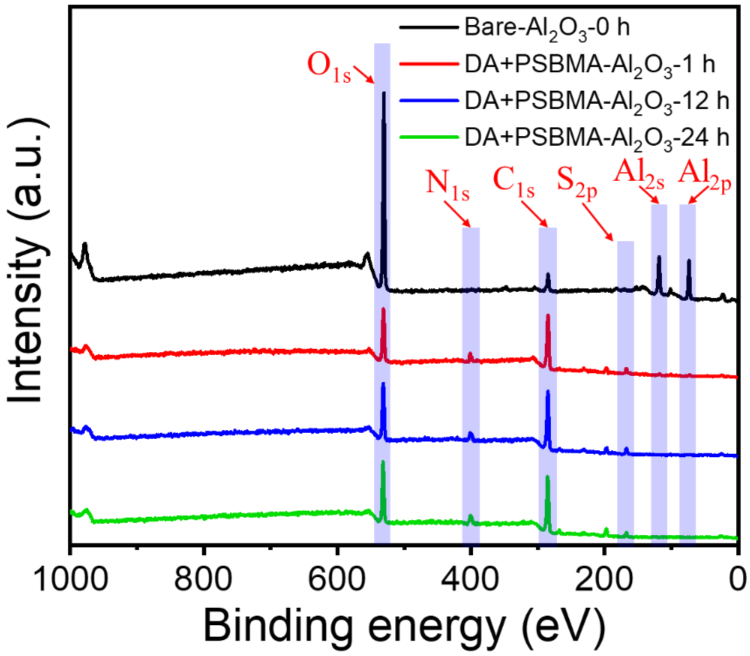

Figure S4. XPS survey spectra of flat DA+PSBMA-Al_2O_3 substrates prepared by co-deposition for 0, 1, 12, and 24 h.

Table S1. XPS results of DA+PSBMA-Al_2O_3 substrates with different deposition times

Co-deposition time period	Atomic ratio[a] (%)					
	C_{1s}	O_{1s}	N_{1s}	S_{2p}	Al_{2p}	Al_{2p} /O_{1s}
DA+PSBMA-Al_2O_3-**0 h**	14.4	62.4	0.9	<0.1	22.3	0.36
DA+PSBMA-Al_2O_3-**1 h**	65.1	26.4	5.5	3.0	<0.1	<0.1
DA+PSBMA-Al_2O_3-**12 h**	64.0	24.3	7.7	2.8	1.1	0.05
DA+PSBMA-Al_2O_3-**24 h**	64.1	26.5	7.7	1.8	<0.1	<0.1

[a]Atomic ratio is obtained by integrating the peak area in the survey XPS spectra, divided by the sensitivity factor of the corresponding element.

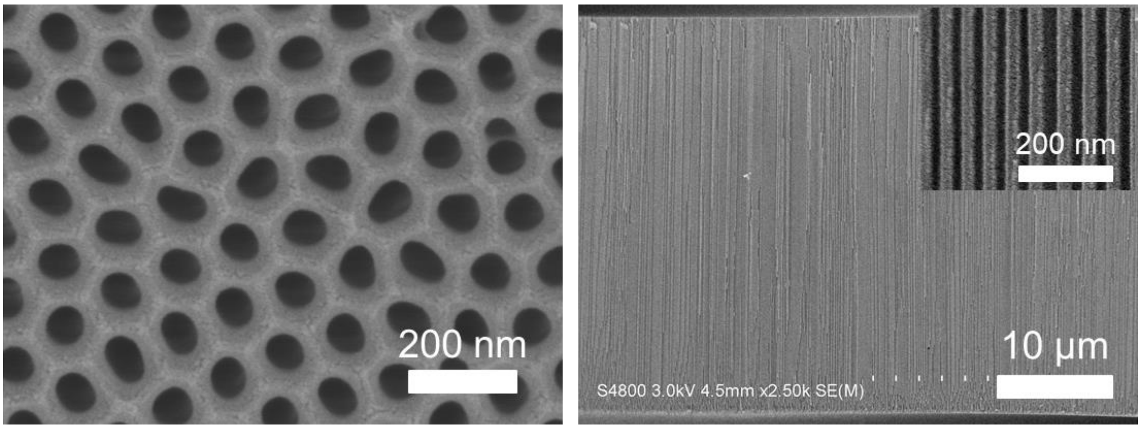

Figure S5. Top-view and cross-sectional SEM images of AAO membranes fabricated with a second anodization time of 2 h, followed by a 30 min pore-widening process. The AAO membrane has a pore size of ~73 nm and a membrane thickness of ~34 μm.

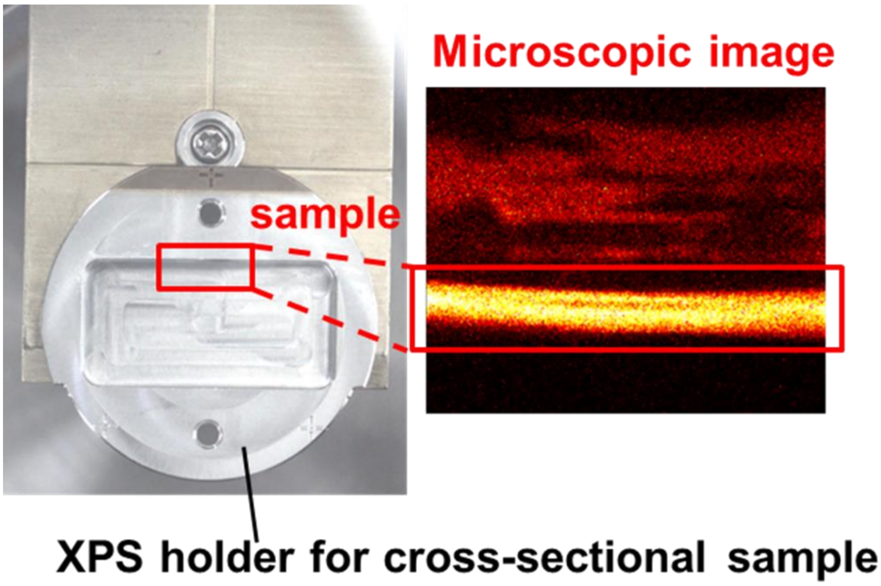

Figure S6. Photo and optical microscopic images showing the setup with a specialized holder for XPS characterization on the cross-sectional surface of the DA+PSBMA-AAO membrane.

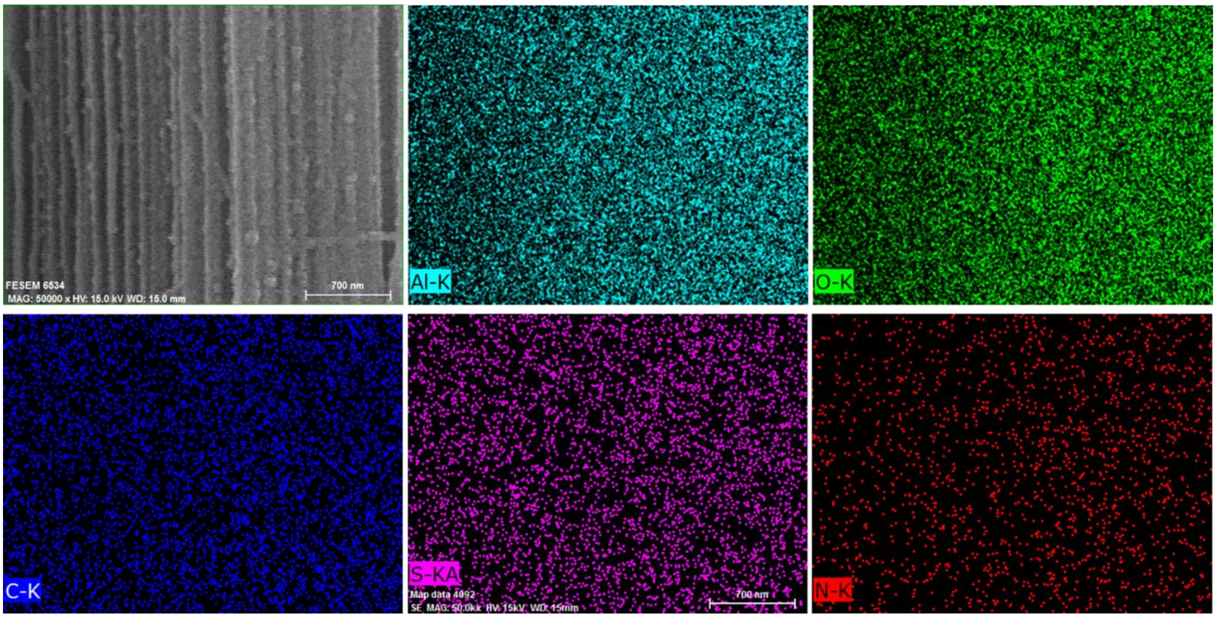

Figure S7. Cross-sectional SEM images of the DA+PSBMA-AAO membrane coupled with EDX mapping on elements of aluminum (light blue), oxygen (green), carbon (blue), sulfur (pink), and nitrogen (red).

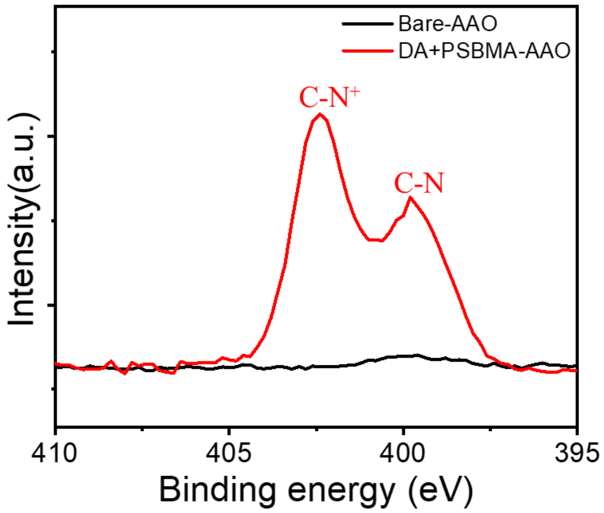

Figure S8. High-resolution XPS spectra obtained from the cross-sectional surface of bare AAO and DA+PSBMA-AAO in N_{1s} region.

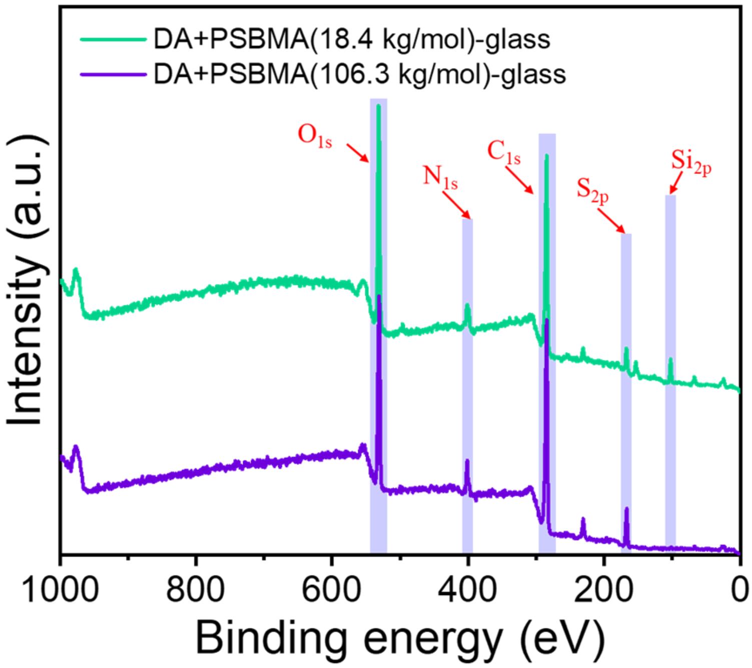

Figure S9. XPS survey spectra of DA+PSBMA coatings after co-deposition for 24 h on flat glass substrates prepared using PSBMA with M_n of 18.4 kg/mol and 106.3 kg/mol.

Table S2. XPS results of DA+PSBMA-glass with different PSBMA molecular weights

M_n of PSBMA	Atomic ratio[a] (%)						MR[b]
	C_{1s}	O_{1s}	N_{1s}	S_{2p}	Si_{2p}	S_{2p}/N_{1s}	(mol/mol)
**PSBMA: 18.4 kg/mol**	59.3	27.5	8.8	1.1	3.3	0.13	0.25
**PSBMA: 106.3 kg/mol**	63.7	25.3	7.0	3.8	0.2	0.54	1.64

[a]Atomic ratio is obtained by integrating the peak area in the survey XPS spectra, divided by the sensitivity factor of the corresponding element. [b]Molar ratio (*MR*) of SBMA/DA on the surface. The *MR* value can be derived from S_{2p}/N_{1s} by assuming that surface composition of the deposited coating is in accordance with that of the inner layer.

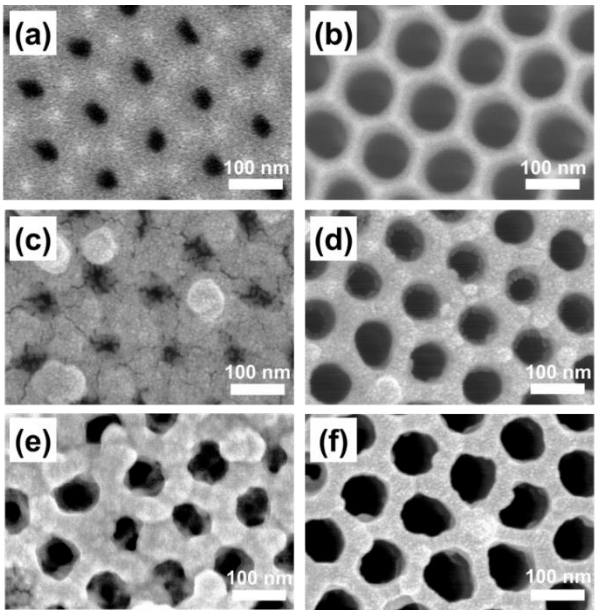

Figure S10. Top-view SEM images of (a–b) bare AAO, (c–d) DA+PSBMA-AAO prepared using PSBMA with M_n of 106.3 kg/mol, (e–f) DA+PSBMA-AAO prepared using PSBMA with M_n of 18.4 kg/mol. The pore sizes of the AAO used in (a), (c), and (e) are ~41 nm, while those in (b), (d), and (f) are ~87 nm.

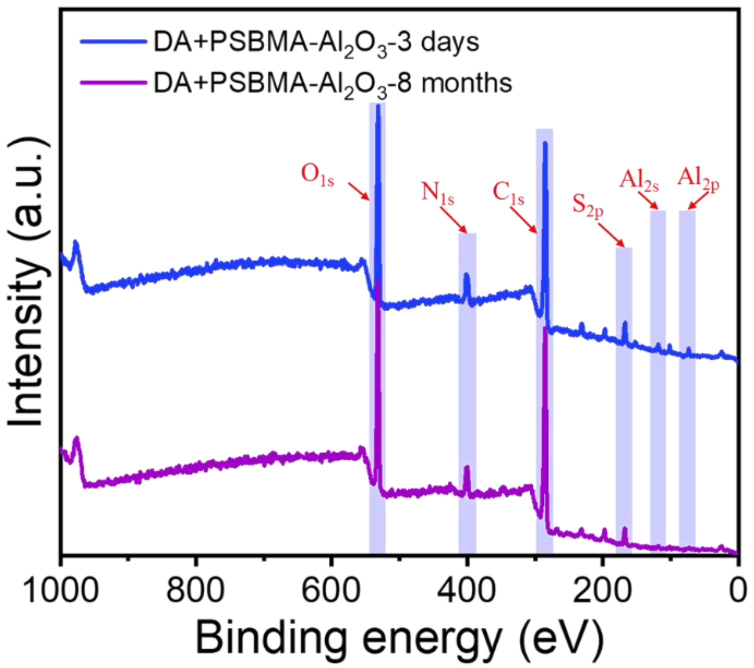

Figure S11. XPS survey spectra of DA+PSBMA-Al_2O_3 stored under air atmosphere for 3 days and 8 months.

Table S3. XPS results of DA+PSBMA-Al_2O_3 stored in air for different time period

Time period stored in air	Atomic ratio[a] (%)					S_{2p} /N_{1s}	*MR*[b] (mol/mol)	Al_{2p} /O_{1s}	*CC*[c] (%)
	C_{1s}	O_{1s}	N_{1s}	S_{2p}	Al_{2p}				
0 days	64.1	26.5	7.7	1.8	<0.1	0.23	0.44/1	0.004	>99
3 days	63.0	26.6	8.2	2.2	<0.1	0.27	0.53/1	0.004	>99
8 months	64.7	28.8	7.1	1.3	0.2	0.18	0.34/1	0.007	99.0

[a]Atomic ratio is obtained by integrating the peak area in the survey XPS spectra, divided by the sensitivity factor of the corresponding element. [b]Molar ratio (*MR*) of SBMA/DA on the surface. [c]Coating coverage (*CC*) value was calculated by $[1-1.5(Al_{2p}/O_{1s})] \times 100$ (%).
